# A landscape framework for an environmental land use ethic

**DOI:** 10.1177/09632719251415432

**Published:** 2026-01-27

**Authors:** Teea Kortetmäki, Yousef Sakieh

**Affiliations:** 1 Department of Social Sciences and Philosophy, University of Jyväskylä, Jyväskylä, Finland; 2 School of Resource Wisdom, University of Jyväskylä, Jyväskylä, Finland

**Keywords:** Human–nature relations, land use, land ethic, landscape, methodology

## Abstract

Land use practices and their expansion raise pressing questions for environmental ethics and have been identified as a key driver of biodiversity loss. In this article, we examine how well the existing environmental ethics and political philosophy approaches suit for addressing the normative questions of land use, especially when empirical knowledge about human land use impacts on nonhuman life is also considered. We point out that the current approaches typically address land use with intactness-, resource-, or integrity-based perspectives. These perspectives form a continuum between quantity-oriented and quality-oriented philosophical approaches to land use. We argue that existing approaches are insufficient for theorizing the environmental ethics of land use: what is needed is an integrative perspective that better captures the constituents of a landscape that human activities might influence. We address this gap and make a theoretical contribution by providing a framework that integrates elements from the existing philosophical approaches with landscape ecology and helps address the quality of land use and land management. This framework helps normative environmental theorizing about land use in a way that is sufficiently nuanced and empirically informed.

## Introduction

New ways to think about the quality of human impact in environmental ethics are needed, as land use by humans^1^ covers more than half of Earth's habitable land. Lands that are in active human use for settlement, agricultural, industrial, or alike purposes have received relatively little attention in environmental ethics and environmental political theory (EE and EPT for short). This neglect is problematic because examining land use in normative environmental theorizing is important for several reasons. First, land use change is empirically confirmed as one of the main drivers of habitat degradation. Thus, land use is a central topic for any ethics concerned with biodiversity loss. Second, significant land use is unavoidable even in the most environmentally considerate future scenarios of human life on Earth. Third, empirical knowledge demonstrates that different land use and management practices influence differently land suitability for different nonhuman life forms. Thus, the central question is not merely whether humans use land, but *how* they use it. More precisely, the EE of land use raises the following key questions:

1. *What* reasons and preconditions justify appropriating land for human uses?
2. *How much* are humans morally permitted to appropriate land for their uses?
3. *Where* should different land uses be located, and according to which guiding norms?
4. *How* to assess the moral rightness of using land (e.g. farming, housing, and urban planning)?

Calls to address the quality of human impact regarding land use practices have been made in EE (e.g. Hettinger, 2002), yet missing are positive answers and elaborate considerations that would help develop environmental land use ethics further to suit different real-world contexts. In this article, we discuss philosophical approaches and their implications and limitations regarding land use and suggest resolutions to the noted shortcomings. We focus on lands that are already in human use, emphasizing the questions of how land is used (this can also be labeled as land management, a sub-topic of land use questions).^2^ We argue that the existing approaches lack tools to handle land use with sufficient breadth. What is missing is a philosophical-theoretical framework for describing land use relative to its impacts on nonhuman life forms and diversity. This requires, we claim, a landscape-informed conceptual framework. Consequently, we construct such a landscape framework and discuss how it helps environmental philosophical theorizing engage in the ethics of land use. Our framework is a theoretically novel contribution in philosophical land use theorizing and supports further work in this domain, both in itself and by demonstrating the translation of ecological knowledge into philosophical use.

Our methodology is interdisciplinary. Understanding the fundamentals of ecological dynamics helps make EE appropriate for real-world contexts. Appropriateness here assumes two things. First, theorizing should reach beyond thinking the quantity of land use and help differentiate various ways of using land to different human purposes as more or less permissible or desirable (cf. Hettinger, 2002). Second, normative theorizing should be empirically informed so that the argumentation is not contradictory or counterintuitive with relation to the key messages that ecology provides. Beyond responding to these demands, we contribute to moral epistemology by discussing the relationship between empirical evidence and the justification of ethical-theoretical beliefs. We, however, do not argue here for a particular normative view: the landscape framework can be applied to many.

The work proceeds as follows. The “Land use: Degrees of human interference” section characterizes predominant meanings that normative environmental theorizing has given to land use. The “Landscape as a socio-ecological construct” section draws on landscape ecology to conceptualize landscape as a key analytical unit for thinking about land use in the fields of EE and EPT (not as a scenery but as a heterogeneous area with various constitutive elements). The “Distinguishing the ‘whether’ and ‘how’ of land use in ethics” section discusses how normative environmental theorizing has conceptualized land use. The suitability of these approaches is evaluated in the “Current approaches to land use in EE and EPT” section. The upshot is that the existing approaches are insufficient for environmental land use ethics due to several shortcomings. To address the shortcomings, “A landscape framework for EE” section constructs a landscape framework that supports normative environmental theorizing of land use.

## Human interference and landscapes

Perceptions and descriptive frameworks influence what ethics can say about entities: a descriptive framework precedes the normative. Different descriptions direct attention to different types of actors, recipients, and impacts or relationships as requiring normative scrutiny. In EE, disciplinary debates about such descriptions concern not only living entities but also their environments. Human-land relations are an example of this.

### Land use: Degrees of human interference

Human activities have left at least some kind of imprint on every place on Earth, either due to active land use practices or due to the impacts of global environmental changes. Environmentalists and environmental philosophers have characterized the situation differently. At extremes are views where nature is considered as lost (McKibben, 2006; Rolston, 2001) or as present even in shopping malls, not much less than in mountains (Vogel, 2015). Both extremes hamper agricultural and urban *environmental* ethics by denying any positive role for humans (Epting, 2017; Hettinger, 2002; Thompson, 1992, 2012) or by permitting unrestricted transformation of the nonhuman world and dismissing the diversity therein (Hailwood, 2015). Finding a middle ground is necessary. Environmental philosophy “needs an ethic for the use of nature, as well as for its nonuse” (Hettinger, 2002: 110) to guide human activities, not only omissions.

Fortunately, nowadays, many views have started to identify differences in how humans are present and interact in the natural world with different entities, which helps avoid extreme characterizations. This diversity is characterized in Table 1 as a typology of the different types and degrees of human interference with the nonhuman world that have received attention in EE and EPT. The list is not exhaustive but captures common examples. Notably, there is dynamism that a table cannot capture: lands do switch between categories, yet the pace of change varies depending on the change direction.

**Table 1.** Common perceptions of the degrees of human interference with land.

	Degree of interference	Examples	Main EE and EPT discussions that link to the examples
Lands where human involvement is not (currently) active	Indirect interference, no human presence, invisible	Climate change impacts in any areas that do not fit into the categories below	Anthropocene (climate change), modernity and industrialization, loss of value, and conservation prospects
	Indirect interference, no human presence, visible	Like above, but visible (seeing may require expertise) impacts, such as Shrinking Alpine glaciers or water body eutrophication	Ecosystem degradation, loss of value and conservation prospects, and harm to nonhuman inhabitants
	Interference with only occasional or very small-scale human presence	Recreational areas (hiking and fishing), and some areas inhabited by indigenous communities	Definitions and loss of wilderness, and conservation prospects
	Direct “ex-interference,” withdrawn presence	Ex-mines, ex-settlements, and ecological restoration areas where active interference has stopped	Ecological restoration and restoration of value
Lands of currently active human involvement	Ongoing interference, occasional human or human-tool presence	Commercial forest, managed pasture, managed recreation areas	Agricultural and forestry environmental ethics, rarely discussed
	Ongoing involvement, frequent human or human-tool presence	Industrial production, most agriculture, active ecological restoration, and very popular recreation areas	Land use ethics, agricultural environmental ethics, rarely discussed
	Ongoing involvement, constant human or human-tool presence	Urban areas, industrial zones	Urban environmental ethics, rarely discussed

EE: environmental ethics; EPT: environmental political theory.

Table 1 demonstrates the variety in the degrees and types of human interference and involvement that may occur. Three things are worth noting. First, excluding some exceptions, an increase in the degree of active human involvement seems to decrease the degree of interest that EE and EPT theorists have shown in the type of human presence and interference with land. Second, the degree of interference or the type of land use does not correlate directly with the negative human impact on nonhuman flourishing and biodiversity. Third, there are temporal dynamics that EE pays quite little attention to.^3^ Consequently, reasoning that makes assumptions about nonhuman flourishing directly based on the degree of human interference or the type of land use are misinformed. More is needed to do EE of land use that shows adequate consideration for the nonhumans’ prospects for flourishing and continued existence in the changing world.

### Landscape as a socio-ecological construct

Landscape ecology considers landscapes as spatially determined, heterogeneous, and temporally evolving areas with human and nonhuman presence: “A landscape can be defined as a perceivable place of living for human and nonhuman beings” (Duflot et al., 2024). Within landscape boundaries, there are often different types of land such as forests, agricultural lands, and urban areas, although landscape analysis can also be scaled down to a single-ecosystem level. Land use diversity brings spatial heterogeneity to the landscape. Within the landscape, these areas interact with each other through the movement of energy, resources, and organisms. Such interactions are driven by natural factors (e.g. topography, climate, and vegetation) and societal factors (e.g. urbanization, agriculture, and forestry), indicating the collective nature of such forces in driving landscape dynamics and shaping the overall character of a landscape. As socially constructed, analytical, and hermeneutic entities, landscapes lack objectively determined boundaries. Boundary definition depends on the purpose for which landscapes are looked at, akin to determining the boundaries of a human community. The idea of heterogeneity already implies that landscapes are usually, although not always, understood as comprising multiple ecosystems. An ecological perspective sets upper and lower limits to the meaningful size of a landscape as an analytical unit.

The slightly varying definitions for landscapes in different sub-disciplinary approaches have in common that landscapes comprise of three types of components (Lepczyk et al., 2008): (1) a spatial component, (2) structure, and (3) functions (and processes). *A spatial component* refers to the physical measurable area of a landscape. Spatially similar areas can, however, differ greatly in terms of the structure and functions of a landscape. These together influence and determine how well various human and nonhuman life forms can inhabit the area or use its resources. In these terms, humans are essentially inhabiting biotic communities, and the two cannot be separated.

*Structure* of a landscape is characterized by both composition and configuration. Compositional attributes indicate the elements, “ingredients,” which influence landscape structure. For example, a landscape with a total area of 100 ha could have the composition of 40 ha forest, 30 ha agriculture, 20 ha rangelands, and 10 ha urban. Second, configurational attributes describe how these “ingredients” are spatially arranged relative to each other in a landscape. For example, 40 ha of forest can be present as one big coherent patch or as several smaller clusters, fragmented by agricultural fields and urban areas.

*Functions*, or ecosystem services from the human-utility viewpoint, are the roles or tasks that landscape structures, resources, and landscape-occupying entities together perform: habitat provisioning, carbon sequestration, and water filtration, for example. They may cause changes in the landscape structure or contribute to maintaining it. Functions emerge from the coexistence of certain system constituents and make a system more than the sum of its parts. Functions result from processes that occur within the landscape through physical, biological, and chemical interactions. Put simply, we can think of functions as “what” a landscape delivers and processes as “how” the landscape performs the functions (e.g. photosynthesis, decomposition, and evapotranspiration). While the mainstream of landscape studies literature defines functions anthropocentrically as benefits that a landscape delivers to human societies, it is possible to consider and describe functions from the perspective of nonhumans too.

EE and EPT have rarely used the notion of landscape in an ecological sense (the cultural and aesthetic meanings are not common either); ecosystems or habitats are more commonly used. As a notable exception, political philosopher Simon Hailwood determines landscape as “a portion of the natural world insofar as it is physically modified by human activity and/or interpreted for human-oriented ends; molded and used, or viewed as malleable and useful, for the sake of human-centered interests and needs” (Hailwood, 2015: 41–42).^4^ For Hailwood, a natural area becomes landscaped when human ideas or purposes are attached to it, regardless of whether this includes touching (while elsewhere, Hailwood's landscape discussion focuses quite explicitly on the materially impactful “humanizing” relation to land)*.* A difference is Hailwood's focus on the human perspective and human interests as the very anchoring point of a landscape concept. From the ecological perspective, in contrast, an area can also be a landscape from the perspective of nonhuman interests (even if we conceptualize only the socially constructed boundaries for a landscape). Additionally, Hailwood does not consider the actual constituents of any landscape. He discusses human relations in terms of their human impacts or qualities (whether we are alienated from nature or not), which is a different focus and does not help EE or political theory to assess land use in ways that relate to nonhuman flourishing and biodiversity in that area.^5^ A more ecologically grounded definition and framework is needed.

### Distinguishing the “whether” and “how” of land use in ethics

Land use choices invoke questions that might be familiar to many as the “sharing versus sparing” debate in conservation biology and ethics (cf. Fischer et al., 2014). Whereas the sparing approach emphasizes spatial aspects, the sharing pays more attention to the structure and functions of any considered landscape and of land use. Human presence as such says very little about impact on the structures and functions of a landscape (or more specifically discussed ecosystems or habitats therein). In ethical terms, the debate captures how land use considerations immediately reach beyond the spatially focused question of how much humans are justified to use land. It is equally relevant to ask *where*, *what for*, and *how* land can or ought (or ought not) to be used. To address these questions plausibly, we maintain that EE and EPT benefit from incorporating the basic conceptualization of landscapes from landscape ecology. This suggestion is grounded in the assumption that spatial facts about land use say very little about whether the landscape is benign or hostile to nonhuman life, its diversity, or certain vulnerable species and their habitats. Attention is needed to the structural impacts of land use and to the functions and processes that may be influenced by human activities. Together with structure, these functions support different species populations and living beings in their co-existence and striving for well-being (e.g. Duflot et al., 2024), and these possibilities are at the heart of environmental land use ethics.

## Current approaches to land use in EE and EPT

We will next characterize the three main EE and EPT perspectives on land use and connect them with the landscape conceptualization. We consider how different EE and EPT perspectives discuss land use, especially in relation to biodiversity as a general proxy for diverse nonhuman flourishing. We refer to works and authors who represent various perspectives, yet this examination is not a review. The focus is on approaches that have mainstreamed, created active theoretical discussion in EE or EPT, and that provide some positive assessment criteria that could guide land use normatively. Below, we call these approaches as intactness-, resource-, and integrity-based. It should be noted that “-based” is not synonymous to “-driven”: our labels denote the standards of different approaches for assessing the normative justifiability of land use. The accounts from which such standards emerge can, however, be driven by other focus points than intactness, resources, or integrity.

### Intactness-based perspectives

EE was in its early decades largely interested in valuing and preserving wilderness and seeking to define pristine sources of such value (e.g. Light and Wellman, 2003). This was an understandable response to the constantly expanding colonial conquering of territories in the US, the home of many early contributions. The focus on pristine sources of value in nature, such as “intactness” or historical authenticity, was linked to human land use impacts, especially in early generation works on ecological restoration. Restoration-aiming involvement was compared to art forgery that can never achieve the value of the “original nature” (Elliot, 1982). Similarly, Holmes Rolston III stated: “the fallacy is to think that a nature allegedly improved by humans is anymore real nature at all” (Rolston, 1991: 370). Beyond the unavoidably declining value, restoration was rejected as malicious: “The re-created natural environment that is the end result of a restoration project is nothing more than an artifact created *for human use*” (Katz, 1992; italics mine). Since these arguments concerned human activities that aim at *improving* the ecological state, human involvement for other reasons is assumably even worse (yet Rolston, 1994, acknowledges that restoration as restitution is possible and perhaps sometimes desirable).

The negative value of human involvement above is expressed in two forms: (1) A proposition that human land use makes things always worse in environmental-ethical terms than human absence, either by distorting their historically significant evolvement or “state of naturalness” that is of foundational value. (2) A more modest claim that even if human presence or inference could do something good in some situations, human-used land can never have the same value as intact land. Against this background, the lack of urban and agricultural matters in early EE is unsurprising and was occasionally criticized (de-Shalit, 1996; Light, 2001). Ecofeminist and decolonial literature also linked wilderness-orientation to what they consider as problematic grounding assumptions that (re)create oppressive dichotomies (human/nature, culture/nature, etc.) or represent too reductionistic descriptions of the world by dismissing important aspects of relatedness, similarity, and difference (e.g. Ladkin, 2005; Ward, 2019). The criticism, however, missed answers to what “post-intactness” in EE would mean for land use.^6^

The above assumptions leave little space for discussing the quality of human involvement, as both Paul B. Thompson and Ian Thompson have noted (Thompson, 1992, 2012). As Ian Thompson (2012: 450) puts it: “a fixation upon nonhumanized environments (supposed wilderness) has, until recently, pushed consideration of landscapes and the built environment to the periphery.” Human involvement appeared so bad that land use ethics reduced to the demand of minimizing land use: for example, condensing humans maximally in cities to protect nature (Naess, 1995). Norms for restricting human involvement have been diverse yet resemblant: deep ecology says humans have the moral right to interfere with nature only to satisfy *vital* human needs (Naess and Sessions, 1986); Paul Taylor's biocentrism classically suggested the main principles of nonmaleficence and noninterference alongside minimum wrong, restitution, and distributive justice for cases when humans anyway interfere with the nonhuman realm (Taylor, 1986).^7^ Consequently, such views adopt a heavily spatial approach to land use questions: the ethics of land use boils down to the question of whether and much humans are justified to use land and how much should be “set aside.” This approach also mirrors the “Half-Earth” proposal to put aside half of Earth's land and seas for nonhuman nature.^8^ In sum, the intactness-based perspectives were basically equipped and interested in dealing only with the spatial aspects of landscapes.

### Resource-based perspectives

Dealing with resource scarcity has been one of the main themes or assumptions in justice theorizing (Wienhues, 2020: Chapter 3), so it is unsurprising that a similar framing has also been extended to the nonhuman realm, especially when speaking of justice to nonhumans. Among the first ecological justice accounts, Brian Baxter (2004) proposed an idea of distributive ecological justice: that organisms can have their fair share of environmental resources, including but not limited to physical space. Spatial area is one constituent of the scarce resources—either the main constituent or one among other goods that may also include material resources and life-supporting ecological functions (Wienhues, 2020: 75–82). Speaking of scarce resources implies thinking of rivalrous goods, the use of which by one party leaves less for others.

A recent, comprehensive treatment of resource scarcity as a matter of global distributive ecological justice comes from Wienhues (2020: 73ff), who proposes ecological space as the metric of biocentric distributive justice. It is comprised of physical space, but also material goods and service-like processes. Ecological space can be used, but also (re)generated (renewable resources) or degraded.^9^ While this broadens the perspective beyond merely asking *how much* ecological space is used, the work represents a resource-based approach. This shows in the suggestion that using and degrading ecological space are “more immediately practically relevant for considerations of distributive justice as they … can be measured, and thus, distributed or ground calls for compensation” (Wienhues, 2020: 84; Chapter 5). The resource perspective broadens land use thinking beyond the spatial component by acknowledging how the spatial size of an area is distinct from its size in terms of ecological space, and how the ecological space of any given area may indeed increase or decrease over time.

The resource-based approach resembles the scarcity-based perspective on conservation and land use. The focus is especially on finite or scarce distributable goods (resources). These resources, often in certain habitats, are the object of preservation, conservation, or other regulation. The resource-based approach may consider some landscape structure elements by catching resource diversity. Material resources and areas are, still, often portrayed as rivalrous goods: their use by one party leaves less to others. For operationalizable concepts of distributive justice, it is practical to define elements that distribution can meaningfully concern, to ensure that material aspects of justice are kept in sight; but this captures only a narrow fragment of what landscape ecology shows relevant for land use.

### Integrity-based perspectives

Perspectives that regard non-distributable land attributes typically focus on integrity, health, or functioning, affected by land use practices. The notions are interlinked. Integrity refers here to the structural and functional understanding of ecological (often ecosystem) integrity. It is also called ecosystem flourishing. Integrity is a state where system-typical self-organizing and self-regenerative capacities and processes enable a living system to continue its existence as the kind of entity it is (Schlosberg, 2007). Integrity allows dynamism, systems change due to system-typical capacities (Crescenzo, 2013; Kortetmäki et al., 2023; Roche and Campagne, 2017). Structural and functional integrity differs from “the integrity of wilderness” (also biological integrity) (Roche and Campagne, 2017), which refers to intactness or the absence of human alteration; this notion has gained attention in conservation ethics (Callicott, 1995; Scoville, 2016).^10^ Ecosystem health and integrity are often described interchangeably; when distinguished, systems may retain health even after having lost integrity (Callicott, 1995). Functions, nevertheless, are constitutive to ecosystems and their integrity (Callicott, 1995). In philosophy, functions are often undefined or defined vaguely as the supporting of biodiversity or of system-inhabiting fauna and flora or as operations that are constitutive to ecosystems (although see e.g. Higgs, 2003, on restoration ethics).

Aldo Leopold's land ethic exemplifies an integrity-based approach. For Leopold, guiding concepts for land ethic assessments include *land health*, that is, land's capacity for self-renewal; *soil fertility*; and *integrity* (Leopold, 1949: 221–225).^11^ These factors that influence the landscape's ability to support nonhuman flourishing represent structural-functional elements in the landscape conceptualization. Empirically, Leopold points out how land reacts differently to human forces in different circumstances and discusses in greater detail, for example, energy flows and landscape structural matters (Leopold, 1949: 206–217). His belief in the possibility of human involvement that benefits nonhuman nature is exemplified in the earlier essay *Farmer as a conservationist* (Leopold, 1992). Discussion about conservation in agricultural landscapes captures Leopold's call for the active role of land management: “Resources may get out of order before they are exhausted, sometimes while they are still abundant. Conservation, therefore, is a positive exercise of skill and insight, not merely a negative exercise of abstinence or caution” (Leopold, 1992: 257).

Integrity-based approaches are common, especially in political theories on ecological or multispecies justice (comprised of distributive, recognition, and procedural justice). Integrity describes conditions that enable ecosystems to continue evolving and functioning systems-typically (Schlosberg, 2007: 147–150). The threshold for injustice is harm done to ecological integrity (Crescenzo, 2013; Schlosberg, 2007). The importance of resilience in maintaining integrity (Crescenzo, 2013; Kortetmäki, 2017) suggests that resilience-supporting land use is more just. Integrity can also concretize how the recognition of nature can be realized in land use via particularizing nature into multiple nonhuman stakeholders with differential integrity-related needs that need consideration in land management (Kortetmäki et al., 2023). However, it often remains unclear what an unjust integrity disruption would be (Wienhues, 2020: 127).

Integrity-oriented approaches are also applied in ecological restoration ethics to characterize the aimed impacts of restorative land management: restored ecological integrity (Karr et al., 2022; Scoville, 2016). Integrity-oriented approaches view non-dominating human-land relations as possible when practices adhere to restoration with determined ethical standards (Attfield, 1994; Ladkin, 2005). The debate, however, employs several meanings of integrity, often without a clear distinction. Criticism for the use of integrity in restoration ethics (Rohwer and Marris, 2021; see Karr et al., 2022 for a response) claims that integrity poorly captures what has value in ecosystems (see also Callicott, 2013, Introduction) and proposes that more nuanced concepts like diversity and complexity suit better suited for distinguishing positive and negative human impacts. Nuancing is another reason for incorporating structural and functional factors into land use ethical considerations. However, as an exceptional land use context, restoration cannot ground the environmental land use ethics for agricultural, industrial, and urban purposes.

## Suitability of the different approaches for the ethics of land use

How well do the above approaches suit developing environmental land use ethics and political theory? First, a comprehensive theory should be able to reason all the key questions of land use ethics, to recapitulate:

1. What reasons and preconditions justify appropriating land for human uses?
2. How much are humans morally permitted to appropriate land for their uses?
3. Where should different land uses be located and according to which guiding norms?
4. How to assess the moral rightness of using land (e.g. farming, housing, and urban planning)?

One notable point is that questions 1–3 require theories that function on multiple scales, which is a shortcoming in current EE (Callicott, 2013). Another aspect is that a well-suited *environmental* theory should open the door for the consideration of nonhuman interests alongside human ones, even if it leaves room for moral anthropocentric and non-anthropocentric accounts. To succeed in this, a theory should be in coherence with the scientific ecological knowledge about the different mechanisms of human impacts on nonhuman life and help considering the main explanatory factors. Finally, a theory should be able to operate on multiple scales and contexts that are relevant in land use.

Below, we will link our assessment of the above approaches with the conceptual framework of landscapes as a litmus test for the coherence with empirical knowledge and for the multi-context applicability. This reminds us how areas of the same spatial size may host very different conditions for nonhuman life. Beyond physical space, all life forms need certain a-/biotic resources (landscape composition), their suitable arrangements (landscape configuration), ecological functions, and interspecies relations (diversity). These attributes only partly relate to the area size.^12^ This concerns both active land use and habitat protection. For example, whether certain types of resources reside in fragmented “pockets” or as highly connected is one of the key factors for species’ continued existence (Duflot et al., 2024). Thus, attention is required for all landscape attributes.

### The suitability of various approaches to theorizing human-managed lands

Intactness-based approaches are not willing to go “inside” land use. They address the key questions 1 and 2. This makes them ill-equipped for dealing adequately with human-managed lands; the ideal of intactness is nonsensical in contexts such as cities or croplands, and applying it there would often create loss-loss-outcomes for both humans and nonhumans. For example, cities tend to create conditions beneficial to a few dominant opportunist species like humans and urban pigeons. Non-interference would support their increasing domination over time. Minimized interference in cropping would likely collapse yield levels without bringing notable benefits to diverse nonhuman flourishing either. Simply put, intactness-based approaches are quite useless for thinking about questions 3 and 4 in environmental land use ethics. EE has, admittedly, evolved much after the peak years of intactness-based discussions. However, given their significance and legacy of the field, one must ask how much intactness-based assumptions still influence almost all EE and EPT by reproducing certain dualistic assumptions where the human presence marks disvalue for nonhuman flourishing and biodiversity. The tendency of EE and EPT to keep reasoning universalizable and avoid too much contextual detail increases the risk of implicitly reproducing canonical assumptions. One mark of the legacy, in our view, is the still scant discussion about urban and agricultural EE.

Resource-scarcity-based perspectives can better address the questions of human-managed lands as they acknowledge that human involvement might have differential impacts on resources. The resource-perspective shifts focus to resource efficiency and land sparing in human practices, helping in answering the key questions 1 and 2, partly 3. However, they fail to consider how two areas with similar resource piles can be very different to nonhuman flourishing due to human impacts and broader landscape-level factors. Looking at resources as rivalrous goods also neglects their spatial diversity and configurational arrangement, whose importance is exemplified by road infrastructure. Despite small impacts on the resource pile, road infrastructure may heavily disrupt the physical connectedness of habitats and be highly harmful to their nonhuman inhabitants. Another dynamic that resource perspective captures poorly is spatial heterogeneity: the uneven distribution of certain goods (including organisms and their populations) across a landscape. Spatial heterogeneity contributes positively to biodiversity in many cases. Yet, the implication of this—suggestion for preferring a partly unequal distribution of resources on commonly considered scales—seems hard for many resource-based approaches: they would need further development to deal with such a call for distributive inequality. Attention to landscape configuration, thus, requires stepping beyond simple resource accounting. Some works take steps in this direction (Wienhues, 2020), yet still focus on distribution. They also need more development to attend to functions that are sometimes more important difference-makers for nonhuman flourishing than resource availability.

Integrity-based perspectives are equipped to consider functional aspects and regard ecosystem functions and processes. Thereby, they enable evaluating circumstances where resource impacts alone do not explain harms or benefits to nonhuman life, helping address questions 3 and 4 and partly question 2 (suggesting that land use must not undermine ecological integrity). Integrity-based perspectives help incorporate interspecies dynamics via looking at functions. This is valuable for two reasons. First, it helps understand why endangered species are not always helped by decreased but by increased human interference: for example, the conservation of traditional rural biotopes is “management-dependent” (Raatikainen et al., 2017). Second, by looking *events* and *unfoldings* (functions and processes) in land use, an integrity-based perspective discloses nonhuman agency, the dismissal of which plagues human-driven landscape and land use conceptualizations like that of cultural landscapes (Plumwood, 2006). However, the notion of integrity easily remains too vague or all-encompassing (cf. Rohwer and Marris, 2021; Wienhues, 2020). Full functioning as the criterion of integrity (Schlosberg, 2007) does not help, without standards, for seeing when the functioning of a system is “full” and why. Thus, integrity-based perspectives are susceptible to misleading interpretations about “full functioning” without reference points to empirical knowledge and greater specificity on structural aspects that also matter to address questions 3 and 4 plausibly. Finally, integrity does not automatically incorporate the resource perspective needed to explain standards for *full* functioning. Diagnoses about integrity harms and compromises (e.g. Kortetmäki et al., 2023; Schlosberg, 2007: 149, 2012) do not explain how the integrity-based perspective should incorporate resources into its thinking.

In sum, resource- and integrity-based perspectives on land use ethics have different merits but also shortcomings. More than their integration is needed: neither provides a sufficient explanatory role for the functions, processes, and configurational elements of a landscape in a comprehensive manner. They also lack tools for conceptualizing the cross-system and cross-scalar impacts of ethical argumentation. New elements are needed to direct attention to aspects that matter for making the ethical examination of land use sit in equilibrium with ecological knowledge. To address these needs, we next introduce the landscape framework to conceptualize the impacts of land use on nonhuman life in different contexts for EE and EPT.

## A landscape framework for EE

A landscape framework for EE integrates valuable elements from the resource- and integrity-based philosophical perspectives, combined with elements drawn from landscape ecology and land use studies. The reason for landscape-framing, rather than speaking of ecosystems or biotic communities (more common to EE), is in matters of scale. A landscape framework can do everything that an ecosystem framework could do because of the conceptual synchrony (remember that a landscape can also comprise a single ecosystem), yet the landscape perspective allows incorporating multiscalar thinking to land use ethics, which is critical to address all key questions for this subfield. Ecosystems always interact with neighboring ecosystems. Many human and nonhuman inhabitants move between ecosystems to obtain their needed resources. Many harmful effects of human actions spill over ecosystems: consider chemical use in farmlands that may impact on peri-urban pollinators or aquatic life in rivers, lakes, or seas. Benefits may spill over, too. Thus, it would be often useful to look at the broader landscape in EE to examine human involvement with land. Yet, attention is required to the ecosystem and habitat levels too: otherwise, there is a risk that nonhuman flourishing is treated as a matter of creating distant “refuges” (land sparing) instead of paying attention to the *quality* of land use and management practices where they happen. A landscape framework can capture all of these. After evaluating the strengths and weaknesses of the existing philosophical approaches to land use and management, we scrutinized how the landscape ecology concepts and knowledge can help address the weaknesses. Through interdisciplinary iterative processing, we then selected the most relevant aspects from landscape ecology and land use studies and brought them together with the valuable evaluative elements from resource- and integrity-based philosophical approaches. The ultimate aim of this iterative process was to organize philosophical and landscape research elements together into a framework that would serve EE for land use.

Below, Table 2 introduces the result of this iteration: a landscape framework that helps to think about human involvement in normative environmental theorizing in an ecologically informed way. The table combines EE and EPT concepts with empirically informed conceptions from landscape ecology and land use studies. These attributes are applicable across scales, making them practical and flexible for different study locations and conditions. The table constituents also address temporal aspects, but discussing them in detail goes beyond the scope of this article.^13^ Table 2 demonstrates how the degree of human involvement is interlinked to the structural and functional diversity of different land uses in diverse ways that together explain the prospects of nonhuman flourishing and biodiversity in these contexts. Its applicative and illustrative values are discussed further after the table.

**Table 2.** A landscape framework for environmental land use ethic.

Landscape type by the dominant land use	Degree of human involvement	Structural diversity	Functional diversity from the viewpoint of nonhuman flourishing and biodiversity
Densely built urban areas (industrial zones)	High	Low **Composition:** Lots of abiotic human-made ingredients: urban infrastructure and sealed surfaces (roads and concrete). **Configuration:** Highly compacted urban areas with high densities; low heterogeneity	Low (e.g. limited habitat provisioning and diversity mostly in the form of refuge habitats, low capacity for climate regulation and soil retention)
Urban areas with green infrastructure, some peri-urban areas (“green cities”)	High	Low to medium **Composition:** Predominantly urban infrastructure but with the presence of green cover and less sealed surfaces. **Configuration:** Moderately connected urban patches and highly fragmented green covers; low-to-medium heterogeneity	Low to medium (e.g. limited habitat diversity, low-to-medium capacity for pollination and nutrient supply)
Homogeneous agricultural landscapes (large-scale monocultures)	High	Low to medium **Composition:** Large monocultural fields, that is, human-composed constellations of uniform vegetation. **Configuration:** Highly compacted crop-fields with large patches, highly fragmented other green covers; low heterogeneity	Low to medium (low-to-medium capacity for pollination, habitat provisioning, nutrient supply, and soil retention)
Biodiversity-supportive agricultural landscapes (agroforestry and organic polycultures)	Low to medium	Medium to high **Composition:** Diverse green cover vegetation and animals habiting the area. **Configuration:** Mixed agricultural and forest areas with physical connectedness and compactness; high heterogeneity	High (e.g. rich habitat provisioning, carbon sequestration, and soil retention)
Unmanaged forest or continuous-cover forestry landscapes	Low	Medium to high **Composition:** Native and managed forest vegetation and forest occupying animals. **Configuration:** Large, contiguous forested areas; medium-to-high heterogeneity	High (e.g. rich habitat provisioning and genetic diversity preservation, carbon sequestration, soil formation, nutrient cycling, and climate regulation)
Mixed landscapes	Low to medium	High **Composition**: mosaic of different land uses such as croplands, pastures, grasslands, slightly managed forests, and so on. **Configuration**: A patchy landscape with diverse clusters of different land use types	High (e.g. habitat provisioning, supporting food webs, pollination, carbon sequestration, genetic diversity, nutrient cycling, microclimate regulation, pest and disease control)

Table 2 does not suggest that all possible aspects in any context should be included in ethical examinations. Rather, it proposes context-specific focus points to direct attention to where human practices make a big difference for nonhuman flourishing *and* biodiversity. Next, we revisit the four key questions in the EE of land use (stated in the “Introduction” section). We discuss how the proposed framework helps in answering them by giving preliminary answers and specifying sufficiently empirically informed directions for further examination in ethics.

*Q1: What reasons and preconditions justify appropriating land for human uses?* A landscape framework suggests a critical precondition for justification: land appropriation can be permitted only when human land uses fit in “as patches in the landscape mosaic” instead of creating large-scale homogeneous ensembles that wipe off the non-anthropogenic features from the landscape. Regarding justified reasons for land appropriation, future research is needed.

*Q2: How much are humans morally permitted to appropriate land for their uses?* The complexity and heterogeneity elements in a landscape framework suggest that a low-to-moderate level of land appropriation can be neutral or even beneficial to the overall diversity by creating additional complexity and heterogeneity in the landscape. The framework, then, does not suggest squeezing human activities into the smallest possible area while maximizing “set-aside areas” for nonhumans as the intactness-based approaches do: instead, it embraces a more integrated and less dichotomic outlook. What counts as “moderate” is scale- and context-dependent, and also depends on the current status of the landscape. This evokes an additional guiding question: would the planned land use support the integrity and heterogeneity of the ecosystem(s) and habitats in the area, and what are its impacts on the connectedness and resilience of different land uses and habitats in the area?

*Q3: Where should different land uses be located and according to which guiding norms?* The composition and configuration perspectives of a land use ethic can be used to generate guiding questions (and even quantitative metrics) to pay attention to various landscape features that are relevant to nonhuman flourishing and impacted by human activities and land use arrangements. A preliminary guiding norm for locating land uses, derived from the framework, would suggest that land use location decisions should strive to sustain the highest possible structural and functional diversity for a landscape. This rejects the monotonous expansion of homogenous human activities, characteristic of the developments in many Western societies, as ethically inappropriate.

*Q4: How to assess the moral rightness of using land (e.g. farming, housing, and urban planning)?* A landscape framework calls attention to the impacts of land use and modification practices on the compositional and configurational attributes of a landscape in order to determine whether human practices are beneficial or harmful to nonhumans at a general level. This helps specify the harm done, evaluate its severity, and consider whether the harm can be prevented, alleviated, or compensated for by some reparative measures. For example, farming tends to reduce the compositional diversity of a landscape. Yet, a farmer can choose improved practices and do restorative measures that compensate for harms and beyond: this way, farmers can even become conservationists by their overall impacts on a landscape, as Leopold (1992) suggests.

As another illustration, we discuss with specific examples how the framework translates into ethical reasoning by providing guiding questions. In urban landscapes, the framework elements translate into questions such as: Is there plant and animal diversity? How can the structural diversity be improved from low to high by, for example, adding green cover and reducing surface sealing? (Structural diversity) Does plant and animal diversity contribute to ecosystem services such as pollination, habitat provisioning, and nutrient cycles? How can capacities and habitat provisioning be improved for different species? (Functional diversity) For the larger, mixed-use landscapes, the perspective scales up. This adds a different perspective to guiding questions. When one looks at a region that includes urban areas, peri-urban areas, and forested and/or agricultural environments, new questions arise. Exemplary questions that the framework gives for such application include: Is there low or high variety in land use types to support species diversity, and how do changes in human activities impact on this variety? (Structural diversity) Does the presence of different land use types support diverse habitat provisioning? Do wildlife corridors support migration and food web relations, including predator-prey dynamics, even in changing environmental conditions? (Functional diversity) Such questions are relevant to both land use professionals and philosophers. Land use ethic, as a topic of further research, should consider these kinds of questions within urban and agricultural EE to demonstrate aspects that are more relevant to human–nonhuman relations than the mere presence or amount of human involvement. Differences between Table 1 and these questions demonstrate the big perspectival shift that the transition from “the amount of human involvement” perspective to “the qualitative impact of human involvement” marks for ethical thinking.

## Conclusion

Environmental ethics of land use “must allow that human beings belong on the planet too, and it must articulate how it is possible for us to respect nature while continuing to be human” (Hettinger, 2002: 114). This requires taking human involvement in certain land areas as the starting point for theorizing. This raises several key questions about whether, when, and how humans are allowed to take land into their use and how the practices of interacting with land should be ethically assessed. To successfully answer these, environmental land use ethic must be ecologically informed (cf. Callicott, 1999; Rozzi et al., 2015) and nuance the human impacts on the nonhuman world better. This will help understand and characterize connections between land quality, land management, ecological relationships, and community dynamics that together explain the impacts of human involvement on nonhuman life forms and biodiversity.

In the intellectual legacy of EE, several perspectives address land use, although often implicitly. Intactness-based perspectives from the early years of EE have little to offer for the questions posed in this article. Resource- and integrity-based perspectives on land use have different valuable elements, but even they typically approach the issue from a perspective that is too narrow. This leads to land use reasoning that might be counterintuitive or to implications that do not conform with environmentally concerned intuitions and may, for example, even lead to action-guiding norms that do not help support biodiversity or nonhuman flourishing in a diverse way. The existing EE and EPT approaches only partially capture aspects that would be needed for theorizing the EE of land use and human–land relations comprehensively.

To address this gap, we create a landscape framework for thinking about land use and related practices in normative environmental theorizing. The framework combines elements from the existing theoretical discussion with a landscape ecology-informed structuring. It describes landscapes as systems where spatial, structural, and functional perspectives are all influential to nonhuman flourishing and biodiversity. This helps EE and EPT address the key questions of the EE of land use in the future to better evaluate the appropriate human way of inhabiting this planet.

## Notes

1. One can think that also nonhumans use land for their purposes. In land use studies, the term “land use” refers explicitly to land use by human agents only. For interdisciplinary cohesion, we stick to this use and speak of (in)habitation and resource use when we think of nonhumans.

2. Land use concerns activities and arrangements that overall determine how much land is used for what human purposes, like agriculture or urbanization. Land management concerns how land use actually happens via different agricultural, forestry, and urban planning practices. Since environmental ethics usually address land use, we stay at this more general conceptual level.

3. Restoration ethics has paid attention to time but restoration as a case of land “use” differs greatly from other intentions and activities of active land use.

4. This differs from cultural landscapes that demonstrate long-term relationship between humans and the environment at a given location, giving a distinguishable human imprint on the area.

5. We do not assume that biodiversity alone would be the sole interest of environmental ethics. Moreover, biodiversity is of course about more than simply species richness.

6. Although environmental ethics is much about values and norms, discussion about these immaterial aspects remains insufficient unless its implications to the material aspects of human life are clarified.

7. Taylor's theory actually gives some guidance on *how* to use land. However, Taylor's biocentrism is very quantity-oriented when it, for example, suggests minimizing the number of killed trees in building a library. Thus, the approach remains intactness-based sidelining the quality (such as the “quality” of killed trees, e.g. their diversity, age, and provisions to other species).

8.
https://eowilsonfoundation.org/what-is-the-half-earth-project/

9. Wienhues also mentions “being ecological space” as the last dimension, referring to how human bodies are ecological space by providing habitat for bacterial species.

10. Biological integrity is also resemblant to organismal intactness (bodily and organismal integrity), discussed in bioethics and medical ethics.

11. Leopold also mentions stability and beauty. Stability is a problematic notion, given dynamism, except for meanings already captured in the notion of integrity. For beauty, the aesthetic-ethical reasoning goes beyond our research scope.

12. The presence of sufficient resources requires a certain amount of space, of course.

13. There is a connection between temporal connectivity and certain types of functions. For example, an urban landscape with high human involvement and longer history of development would often have high temporal connectivity and low internalization of nonhuman life functions. In contrast, protected areas with a long, continuous history would be characterized not only by high temporal connectivity but also greater internalization of functions supporting nonhuman life forms (Santangeli et al., 2023).
